# Elevation of the Subclavian Artery above the Clavicle, in a Phthisical Patient

**Published:** 1860-04

**Authors:** 


*Elevation of the Subclavian Artery above the Clavicle, in a Phthisical Patient.*—There are many circumstances which influence the position of the subclavian artery in the neck; and according to its elevation on a level with or even above the clavicle, so can it readily be tied when necessity demands such a proceeding at the hands of the surgeon. As we are in the habit of encountering this vessel clinically, it is usually situated below or at any rate not higher than the level of the clavicle; its deviation from this, however, is a fact worthy of note and of record. On a reference to Mr. Quain's great work on the Arteries, we find the relative position of the artery and clavicle noted in 25 cases, and the results were as follows:—the artery was not higher than the clavicle in 6; was slightly higher in 11; was half an inch and not exceeding one inch higher in 4. and more than one inch and not exceeding one inch and a half in 4. As may be imagined, the position of the shoulders and the development of the tissues materially influence the position of the artery. It is very possible in lean subjects with long and slender necks, that the artery may oftner rise above the clavicle than we are aware of.

We had the opportunity lately of examining a phthisical patient in St. Bartholomew's Hospital, under Dr. Farre's care, who is much emaciated from his disease, there being already a cavity under his right collar bone, with pectoriloquy and all the usual physical signs of phthisis. His age is thirty-nine, and the disease has existed twelve months, with occasional attacks of hæmoptysis, as we learn from Mr. Schollick, Dr. Farre's clinical clerk. The subclavian artery can be felt almost subcutaneously beating much above the clavicle, and very slight pressure causes pain. There is irritability of the muscles of the neck, and he has much laryngeal irritation as well, possibly ulceration of his mucuous membrane in some part of the larynx below the vocal cords—we say below because the voice as yet is not materially affected. His uvula was elongated, but truncation afforded considerable relief. There is no similar elevation of the subclavian of the left side, which is perhaps peculiar, but possibly depending upon the absence of extensive disease of the lung. The relative position of the important artery and the clavicle is of some value clinically, and has not hitherto attracted much attention, at least among physicians. We are the more anxious, therefore, to bring it before the notice of our readers, and shall not fail to record any well-marked instances of it similar to this case. The patient in the present instance has a pigeon shaped chest, and his clavicles seem depressed.—*London Lancet.*

